# Tetra­potassium dianti­mony(III) tin(IV) tetra­deca­fluoride

**DOI:** 10.1107/S1600536808012865

**Published:** 2008-05-07

**Authors:** A. V. Gerasimenko, E. B. Merkulov, T. I. Usol’tseva

**Affiliations:** a Institute of Chemistry FEB RAS, 159 prospekt 100-letiya Vladivostoka, Vladivostok 690022, Russian Federation

## Abstract

The title compound, K_4_Sb_2_SnF_14_, is built from anionic layers, with an overall composition of [Sb_2_SnF_14_]^4−^ extending parallel to the *ac* plane, and K^+^ cations. The layers are made up from vertex-sharing centrosymmetric SnF_6_ octa­hedra and Sb_2_F_12_ dimers. The Sn—F distances are in the range 1.9581 (14)–1.9611 (17) Å. The Sb polyhedra contain three short terminal Sb—F bonds [1.9380 (14)–2.0696 (15) Å], one short bridging bond [2.0609 (17) Å], one bridging bond of medium length [2.7516 (15) Å], and two longer bridging bonds [3.0471 (18) and 3.117 (2) Å]. The K^+^ ions are coordinated by F atoms with coordination numbers 10 and 8, and K—F bond lengths are in the range 2.6235 (16)–3.122 (2) Å.

## Related literature

For related literature, see: Blatov (2004 ▶); Davidovich & Zemnukhova (1975 ▶); Gillespie (1970 ▶); Kriegsmann & Kessler (1962 ▶); Serezhkin *et al.* (1997 ▶).

## Experimental

### 

#### Crystal data

K_4_Sb_2_SnF_14_
*M* *_r_* = 784.59
Triclinic, 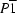

*a* = 6.7356 (2) Å
*b* = 7.4704 (2) Å
*c* = 7.6370 (2) Å
α = 92.691 (1)°
β = 91.461 (1)°
γ = 115.323 (1)°
*V* = 346.53 (2) Å^3^
*Z* = 1
Mo *K*α radiation
μ = 7.00 mm^−1^
*T* = 298 (2) K
0.4 × 0.35 × 0.28 mm

#### Data collection

Bruker SMART 1000 CCD area-detector diffractometer
Absorption correction: Gaussian (*XPREP* and *SADABS*; Bruker, 2003 ▶) *T*_min_ = 0.136, *T*_max_ = 0.291
7147 measured reflections
4635 independent reflections
4250 reflections with *I* > 2σ(*I*)
*R*_int_ = 0.024

#### Refinement

*R*[*F* ^2^ > 2σ(*F* ^2^)] = 0.037
*wR*(*F* ^2^) = 0.090
*S* = 1.30
4635 reflections
98 parameters
Δρ_max_ = 1.91 e Å^−3^
Δρ_min_ = −3.69 e Å^−3^

### 

Data collection: *SMART* (Bruker, 1998 ▶); cell refinement: *SAINT* (Bruker, 2003 ▶); data reduction: *SAINT*; program(s) used to solve structure: *SHELXTL* (Sheldrick, 2008 ▶); program(s) used to refine structure: *SHELXTL*; molecular graphics: *XP* in *SHELXTL*; software used to prepare material for publication: *publCIF* (Westrip, 2008 ▶).

## Supplementary Material

Crystal structure: contains datablocks I, global. DOI: 10.1107/S1600536808012865/fi2062sup1.cif

Structure factors: contains datablocks I. DOI: 10.1107/S1600536808012865/fi2062Isup2.hkl

Additional supplementary materials:  crystallographic information; 3D view; checkCIF report

## Figures and Tables

**Table 1:** Selected bond lengths (Å)

Sb—F1	1.9380 (14)
Sb—F3	1.9539 (13)
Sb—F4	2.0609 (17)
Sb—F2	2.0696 (15)
Sb—F4^i^	2.7516 (15)
Sb—F5^ii^	3.0471 (18)
Sb—F6	3.117 (2)
Sn—F5	1.9581 (14)
Sn—F7	1.9611 (17)
Sn—F6	1.9611 (16)
K1—F4^iv^	2.6235 (16)
K1—F2	2.7086 (14)
K1—F1^v^	2.7268 (16)
K1—F6^vi^	2.811 (2)
K1—F3^iv^	2.8390 (18)
K1—F5^iv^	2.8578 (16)
K1—F1	2.9262 (15)
K1—F3	2.9485 (18)
K1—F4^vi^	2.982 (2)
K1—F7^vi^	3.122 (2)
K2—F2^vii^	2.6662 (15)
K2—F2^v^	2.6777 (18)
K2—F3^iv^	2.7136 (15)
K2—F1	2.7216 (15)
K2—F7^viii^	2.7620 (19)
K2—F7^ix^	2.8795 (17)
K2—F6^i^	2.8943 (16)
K2—F5^iv^	2.9912 (18)

Symmetry codes: (i) 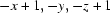; (ii) 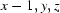; (iii) 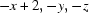; (iv) 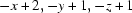; (v) 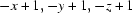; (vi) 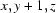; (vii) 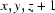; (viii) 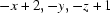; (ix) 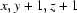.

**Fig. 1.**
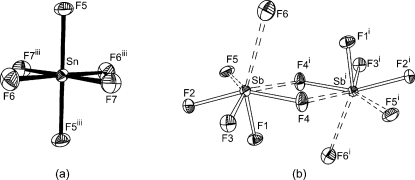
(a) the Sn and (b) Sb coordination polyhedra, with displacement ellipsoids drawn at the 50% probability level. Symmetry codes are given in Table 1.

**Fig. 2.**
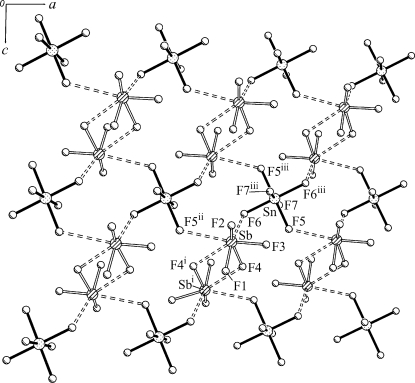
Fragment of the anionic layer [Sb2SnF14]4- viewed along the b axis.

**Fig. 3.**
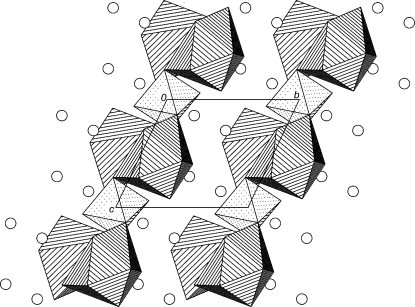
The structure of (I), viewed along the a axis. Sn octahedra are shown as dotted, Sb dimers as parallel lines and K atoms as white spheres.

**Fig. 4.**
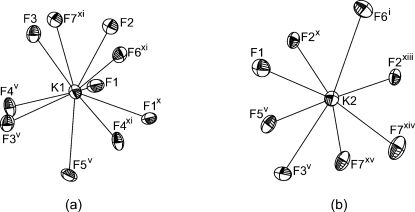
(a) the K1 and (b) K2 coordination polyhedra, with displacement ellipsoids drawn at the 50% probability level. Symmetry codes are given in Table 1.

## supplementary crystallographic information

### Comment

The asymmetric unit of the title compound, K_4_Sb_2_SnF_14_, (I), contains one crystallographically independent Sb atom, one Sn atom on a special position (Wyckoff position 1a), seven fluorine atoms and two potassium cations. The Sn atoms are coordinated by six F atoms in a centrosymmetric, slightly distorted octahedral environment (Fig. 1a), with Sn—F distances ranging from 1.9581 (14) to 1.9611 (17) Å (Table 1). The nearest environment of the Sb atom is formed by  four fluorine atoms with Sb—F bond distances ranging from 1.9380 (14) to 2.0696 (15) Å. Taking into account a lone electron pair (E), the coordination polyhedron of Sb(III) can be described as a trigonal bipyramid (Gillespie, 1970). Three fluorine atoms, F4^i^, F5^ii^ and F6, are involved in the second coordination sphere of Sb with Sb—F bond distances of 2.7516 (15), 3.0471 (18) and 3.117 (2) Å, respectively. Two Sb polyhedra are linked by double fluorine bridges (F4 and F4^i^) to form a centrosymmetric dimer (Fig. 1 b), with an Sb···Sb^i^ distance of 3.9925 (2) Å.

The Sn(IV) and Sb(III) complexes are bound via fluorine bridges yielding the anionic layers [Sb_2_SnF_14_]^4-^ parallel to the *ac *plane (Fig. 2), with Sn···Sb distances of 4.21660 (15) and 4.33908 (16) Å. Such layers alternate with layers of potassium cations (Fig.3).

The coordination numbers (CN) of the potassium cations were calculated by the method of intersecting spheres (Serezhkin *et al.*, 1997) with use of the program package *TOPOS* (Blatov, 2004). For the K1 (Fig. 4a), the CN is 10 [K1—F, 2.6235 (16) – 3.122 (2) Å] and for the K2 (Fig. 4 b), the CN is 8 [K2—F, 2.6662 (15) – 2.9912 (18) Å].

### Experimental

KSbF_4_ (5.94 g, 0.025 mol) was reacted with K_2_SnF_6_.H_2_O (8.21 g, 0.025 mol) in a solution of hydrofluoric acid (10%, 200 ml; Reachem(Russia), 99.99% purity). The solution obtained was evaporated in air at room temperature down to 1/3 of the initial volume. Then the precipitate was separated from solution and dried to constant weight (1.98 g). The complexes KSbF_4_ and K_2_SnF_6_.H_2_O were prepared according to (Davidovich & Zemnukhova, 1975; Kriegsmann & Kessler, 1962).

### Refinement

The maximum peak and deepest hole are located 0.70 Å and 0.96 Å, respectively, from Sn.

### Crystal data

**Table:**

K_4_Sb_2_SnF_14_	*Z* = 1
*M**_r_* = 784.59	*F*_000_ = 354
Triclinic, *P*1	*D*_x_ = 3.760 Mg m^−^^3^
*a* = 6.7356 (2) Å	Mo *K*α radiation λ = 0.71073 Å
*b* = 7.4704 (2) Å	Cell parameters from 2472 reflections
*c* = 7.6370 (2) Å	θ = 3.4–46.9º
α = 92.691 (1)º	µ = 7.00 mm^−^^1^
β = 91.461 (1)º	*T* = 298 (2) K
γ = 115.323 (1)º	Prism, colourless
*V* = 346.526 (17) Å^3^	0.4 × 0.35 × 0.28 mm

### Data collection

**Table:**

Bruker SMART 1000 CCD area-detector diffractometer	4635 independent reflections
Radiation source: fine-focus sealed tube	4250 reflections with *I* > 2σ(*I*)
Monochromator: graphite	*R*_int_ = 0.024
Detector resolution: 8.33 pixels mm^-1^	θ_max_ = 47.0º
*T* = 298(2) K	θ_min_ = 3.4º
φ and ω scans	*h* = −9→13
Absorption correction: gaussian(XPREP and SADABS; Bruker, 2003)	*k* = −15→9
*T*_min_ = 0.136, *T*_max_ = 0.291	*l* = −15→11
7147 measured reflections	

### Refinement

**Table:**

Refinement on *F*^2^	Secondary atom site location: difference Fourier map
Least-squares matrix: full	*w* = 1/[σ^2^(*F*_o_^2^) + (0.0258*P*)^2^ + 0.7101*P*] where *P* = (*F*_o_^2^ + 2*F*_c_^2^)/3
*R*[*F*^2^ > 2σ(*F*^2^)] = 0.037	(Δ/σ)_max_ = 0.003
*wR*(*F*^2^) = 0.090	Δρ_max_ = 1.91 e Å^−^^3^
*S* = 1.30	Δρ_min_ = −3.69 e Å^−^^3^
4635 reflections	Extinction correction: SHELXTL (Sheldrick, 2008), Fc^*^=kFc[1+0.001xFc^2^λ^3^/sin(2θ)]^-1/4^
98 parameters	Extinction coefficient: 0.0782 (13)
Primary atom site location: structure-invariant direct methods	

### Special details

**Table:**

Geometry. All e.s.d.'s (except the e.s.d. in the dihedral angle between two l.s. planes) are estimated using the full covariance matrix. The cell e.s.d.'s are taken into account individually in the estimation of e.s.d.'s in distances, angles and torsion angles; correlations between e.s.d.'s in cell parameters are only used when they are defined by crystal symmetry. An approximate (isotropic) treatment of cell e.s.d.'s is used for estimating e.s.d.'s involving l.s. planes.
Refinement. Refinement of *F*^2^ against ALL reflections. The weighted *R*-factor *wR* and goodness of fit *S* are based on *F*^2^, conventional *R*-factors *R* are based on *F*, with *F* set to zero for negative *F*^2^. The threshold expression of *F*^2^ > σ(*F*^2^) is used only for calculating *R*-factors(gt) *etc*. and is not relevant to the choice of reflections for refinement. *R*-factors based on *F*^2^ are statistically about twice as large as those based on *F*, and *R*- factors based on ALL data will be even larger.

### Fractional atomic coordinates and isotropic or equivalent isotropic displacement parameters (Å^2^)

**Table:**

	*x*	*y*	*z*	*U*_iso_*/*U*_eq_	
Sb	0.611210 (19)	0.191057 (16)	0.312309 (15)	0.01531 (2)	
Sn	1.0000	0.0000	0.0000	0.01467 (3)	
K1	0.85162 (7)	0.73682 (7)	0.41457 (6)	0.02301 (9)	
K2	0.71347 (7)	0.44513 (8)	0.85599 (6)	0.02309 (8)	
F1	0.5699 (2)	0.3363 (2)	0.51424 (18)	0.0247 (3)	
F2	0.5990 (2)	0.4270 (2)	0.18860 (19)	0.0237 (3)	
F3	0.9214 (2)	0.3796 (2)	0.3294 (2)	0.0252 (3)	
F4	0.7266 (2)	0.0671 (2)	0.5021 (2)	0.0300 (3)	
F5	1.1417 (3)	0.1313 (2)	0.22704 (19)	0.0297 (3)	
F6	0.7209 (3)	−0.1279 (3)	0.1151 (2)	0.0332 (4)	
F7	1.0620 (3)	−0.2265 (2)	0.0506 (2)	0.0322 (3)	

### Atomic displacement parameters (Å^2^)

**Table:**

	*U*^11^	*U*^22^	*U*^33^	*U*^12^	*U*^13^	*U*^23^
Sb	0.01754 (4)	0.01469 (4)	0.01477 (4)	0.00794 (3)	0.00034 (3)	0.00109 (3)
Sn	0.01815 (5)	0.01411 (5)	0.01377 (5)	0.00878 (4)	0.00109 (4)	0.00153 (4)
K1	0.01943 (14)	0.02305 (16)	0.02459 (16)	0.00756 (12)	−0.00196 (13)	0.00015 (13)
K2	0.02202 (15)	0.02791 (16)	0.02086 (15)	0.01186 (12)	0.00175 (13)	0.00452 (13)
F1	0.0254 (5)	0.0286 (5)	0.0191 (5)	0.0108 (4)	0.0045 (4)	−0.0032 (4)
F2	0.0302 (5)	0.0201 (4)	0.0233 (5)	0.0131 (4)	−0.0020 (5)	0.0048 (4)
F3	0.0174 (4)	0.0274 (6)	0.0294 (6)	0.0079 (4)	0.0038 (4)	0.0046 (5)
F4	0.0215 (5)	0.0312 (6)	0.0363 (7)	0.0093 (5)	−0.0031 (5)	0.0159 (5)
F5	0.0381 (6)	0.0344 (6)	0.0195 (5)	0.0197 (5)	−0.0083 (5)	−0.0074 (5)
F6	0.0254 (6)	0.0395 (8)	0.0323 (7)	0.0105 (5)	0.0098 (5)	0.0086 (6)
F7	0.0449 (7)	0.0240 (5)	0.0353 (7)	0.0226 (5)	−0.0084 (6)	0.0009 (5)

### Geometric parameters (Å, °)

**Table:**

Sb—F1	1.9380 (14)	K2—F2^xi^	2.6777 (18)
Sb—F3	1.9539 (13)	K2—F3^v^	2.7136 (15)
Sb—F4	2.0609 (17)	K2—F1	2.7216 (15)
Sb—F2	2.0696 (15)	K2—F7^xv^	2.7620 (19)
Sb—F4^i^	2.7516 (15)	K2—F7^xvi^	2.8795 (17)
Sb—F5^ii^	3.0471 (18)	K2—F6^i^	2.8943 (16)
Sb—F6	3.117 (2)	K2—F5^v^	2.9912 (18)
Sb—F7^iii^	3.5325 (19)	K2—F6^xvi^	3.655 (2)
Sb—K1	3.7207 (5)	K2—Sn^xvi^	3.8388 (5)
Sb—F6^iv^	3.8032 (18)	K2—F4	3.847 (2)
Sb—F5	3.8531 (19)	K2—K1^xi^	3.9388 (7)
Sb—F1^i^	3.9018 (16)	K2—F3^xiv^	3.9710 (17)
Sb—K1^v^	3.9464 (5)	K2—Sb^xiv^	3.9808 (5)
Sb—K2^vi^	3.9808 (5)	K2—K2^xvii^	3.9938 (10)
Sb—Sb^i^	3.9925 (2)	K2—K2^xviii^	4.1352 (10)
Sn—F5	1.9581 (14)	K2—F1^xi^	4.1386 (18)
Sn—F5^iii^	1.9581 (14)	K2—K1^v^	4.2602 (8)
Sn—F7^iii^	1.9611 (17)	K2—Sb^xi^	4.3692 (6)
Sn—F7	1.9611 (17)	F1—K1^xi^	2.7268 (16)
Sn—F6^iii^	1.9611 (16)	F1—Sb^i^	3.9018 (16)
Sn—F6	1.9611 (16)	F1—K2^xi^	4.1386 (18)
Sn—K1^vii^	3.7354 (5)	F1—Sb^xi^	4.3632 (17)
Sn—K1^viii^	3.7354 (5)	F2—K2^vi^	2.6662 (15)
Sn—K2^ix^	3.8388 (5)	F2—K2^xi^	2.6777 (18)
Sn—K2^v^	3.8388 (5)	F3—K2^v^	2.7136 (14)
Sb—Sn	4.21660 (15)	F3—K1^v^	2.8390 (18)
Sn—Sb^iii^	4.21661 (15)	F3—K2^vi^	3.9710 (17)
Sn—Sb^iv^	4.33908 (16)	F3—Sb^v^	4.2172 (14)
Sn—Sb^x^	4.33908 (16)	F4—K1^v^	2.6235 (15)
K1—F4^v^	2.6235 (16)	F4—Sb^i^	2.7516 (15)
K1—F2	2.7086 (14)	F4—K1^viii^	2.982 (2)
K1—F1^xi^	2.7268 (16)	F5—K1^v^	2.8578 (16)
K1—F6^xii^	2.811 (2)	F5—K2^v^	2.9912 (18)
K1—F3^v^	2.8390 (18)	F5—Sb^x^	3.0471 (18)
K1—F5^v^	2.8578 (16)	F5—K1^viii^	3.1882 (17)
K1—F1	2.9262 (15)	F5—Sb^xv^	4.9869 (18)
K1—F3	2.9485 (18)	F6—K1^viii^	2.811 (2)
K1—F4^xii^	2.982 (2)	F6—K2^i^	2.8943 (16)
K1—F7^xii^	3.122 (2)	F6—K2^ix^	3.655 (2)
K1—F5^xii^	3.1882 (17)	F6—Sb^iv^	3.8032 (18)
K1—K1^xiii^	3.7178 (9)	F6—Sb^i^	4.9129 (17)
K1—Sn^xii^	3.7354 (5)	F6—Sb^viii^	5.124 (2)
K1—K2^xi^	3.9388 (7)	F7—K2^xv^	2.7620 (19)
K1—Sb^v^	3.9464 (5)	F7—K2^ix^	2.8795 (16)
K1—Sb^xi^	4.0028 (5)	F7—K1^viii^	3.1222 (19)
K1—K2	4.0117 (7)	F7—Sb^iii^	3.5325 (19)
K1—K2^v^	4.2602 (8)	F7—Sb^x^	4.0712 (16)
K1—Sb^xii^	4.4308 (6)	F7—Sb^viii^	4.6870 (16)
K2—F2^xiv^	2.6662 (15)		
			
F1—Sb—F3	87.64 (6)	Sn^xii^—K1—K2^v^	70.956 (11)
F1—Sb—F4	82.57 (7)	K2^xi^—K1—K2^v^	110.414 (15)
F3—Sb—F4	80.01 (6)	Sb^v^—K1—K2^v^	64.623 (11)
F1—Sb—F2	80.77 (6)	Sb^xi^—K1—K2^v^	170.056 (15)
F3—Sb—F2	79.13 (6)	K2—K1—K2^v^	106.442 (15)
F4—Sb—F2	153.71 (6)	F4^v^—K1—Sb^xii^	105.98 (4)
F1—Sb—F4^i^	71.19 (6)	F2—K1—Sb^xii^	103.48 (4)
F3—Sb—F4^i^	143.84 (6)	F1^xi^—K1—Sb^xii^	60.65 (4)
F4—Sb—F4^i^	68.69 (6)	F6^xii^—K1—Sb^xii^	44.34 (4)
F2—Sb—F4^i^	123.67 (6)	F3^v^—K1—Sb^xii^	142.69 (4)
F1—Sb—F5^ii^	80.34 (5)	F5^v^—K1—Sb^xii^	83.36 (4)
F3—Sb—F5^ii^	145.85 (6)	F1—K1—Sb^xii^	123.89 (4)
F4—Sb—F5^ii^	129.10 (5)	F3—K1—Sb^xii^	155.13 (3)
F2—Sb—F5^ii^	67.49 (5)	F4^xii^—K1—Sb^xii^	23.27 (3)
F4^i^—Sb—F5^ii^	60.42 (5)	F7^xii^—K1—Sb^xii^	92.88 (4)
F1—Sb—F6	156.26 (6)	F5^xii^—K1—Sb^xii^	58.05 (4)
F3—Sb—F6	91.23 (6)	K1^xiii^—K1—Sb^xii^	57.132 (15)
F4—Sb—F6	73.89 (6)	Sb—K1—Sb^xii^	132.635 (13)
F2—Sb—F6	122.29 (5)	Sn^xii^—K1—Sb^xii^	61.479 (8)
F4^i^—Sb—F6	96.84 (5)	K2^xi^—K1—Sb^xii^	63.210 (12)
F5^ii^—Sb—F6	112.19 (4)	Sb^v^—K1—Sb^xii^	127.696 (11)
F1—Sb—F7^iii^	144.50 (5)	Sb^xi^—K1—Sb^xii^	56.236 (8)
F3—Sb—F7^iii^	60.78 (5)	K2—K1—Sb^xii^	121.076 (15)
F4—Sb—F7^iii^	105.41 (6)	K2^v^—K1—Sb^xii^	132.216 (14)
F2—Sb—F7^iii^	77.82 (5)	F2^xiv^—K2—F2^xi^	83.28 (5)
F4^i^—Sb—F7^iii^	144.19 (5)	F2^xiv^—K2—F3^v^	138.86 (5)
F5^ii^—Sb—F7^iii^	116.08 (4)	F2^xi^—K2—F3^v^	116.46 (5)
F6—Sb—F7^iii^	49.35 (4)	F2^xiv^—K2—F1	145.86 (5)
F1—Sb—K1	51.20 (4)	F2^xi^—K2—F1	75.98 (5)
F3—Sb—K1	51.92 (5)	F3^v^—K2—F1	75.22 (4)
F4—Sb—K1	108.38 (5)	F2^xiv^—K2—F7^xv^	85.87 (5)
F2—Sb—K1	45.52 (4)	F2^xi^—K2—F7^xv^	163.17 (5)
F4^i^—Sb—K1	121.51 (4)	F3^v^—K2—F7^xv^	80.02 (5)
F5^ii^—Sb—K1	97.07 (3)	F1—K2—F7^xv^	107.02 (6)
F6—Sb—K1	140.19 (3)	F2^xiv^—K2—F7^xvi^	73.56 (5)
F7^iii^—Sb—K1	94.15 (3)	F2^xi^—K2—F7^xvi^	103.37 (5)
F1—Sb—F6^iv^	119.92 (6)	F3^v^—K2—F7^xvi^	67.03 (5)
F3—Sb—F6^iv^	118.94 (5)	F1—K2—F7^xvi^	137.34 (5)
F4—Sb—F6^iv^	149.10 (6)	F7^xv^—K2—F7^xvi^	85.75 (5)
F2—Sb—F6^iv^	56.92 (5)	F2^xiv^—K2—F6^i^	69.80 (5)
F4^i^—Sb—F6^iv^	97.15 (4)	F2^xi^—K2—F6^i^	68.66 (5)
F5^ii^—Sb—F6^iv^	46.60 (4)	F3^v^—K2—F6^i^	149.54 (5)
F6—Sb—F6^iv^	81.11 (5)	F1—K2—F6^i^	77.45 (5)
F7^iii^—Sb—F6^iv^	69.52 (4)	F7^xv^—K2—F6^i^	95.51 (6)
K1—Sb—F6^iv^	102.43 (3)	F7^xvi^—K2—F6^i^	143.13 (5)
F1—Sb—F5	124.91 (5)	F2^xiv^—K2—F5^v^	105.63 (5)
F3—Sb—F5	47.69 (6)	F2^xi^—K2—F5^v^	62.58 (5)
F4—Sb—F5	62.29 (5)	F3^v^—K2—F5^v^	61.36 (5)
F2—Sb—F5	112.55 (5)	F1—K2—F5^v^	88.59 (5)
F4^i^—Sb—F5	123.65 (5)	F7^xv^—K2—F5^v^	133.22 (5)
F5^ii^—Sb—F5	154.74 (5)	F7^xvi^—K2—F5^v^	56.44 (5)
F6—Sb—F5	44.86 (4)	F6^i^—K2—F5^v^	131.19 (6)
F7^iii^—Sb—F5	43.91 (4)	F2^xiv^—K2—F6^xvi^	57.19 (4)
K1—Sb—F5	99.51 (3)	F2^xi^—K2—F6^xvi^	58.00 (4)
F6^iv^—Sb—F5	110.73 (4)	F3^v^—K2—F6^xvi^	101.14 (4)
F1—Sb—F1^i^	101.60 (5)	F1—K2—F6^xvi^	126.75 (5)
F3—Sb—F1^i^	119.88 (6)	F7^xv^—K2—F6^xvi^	124.94 (5)
F4—Sb—F1^i^	43.93 (5)	F7^xvi^—K2—F6^xvi^	47.89 (5)
F2—Sb—F1^i^	160.78 (5)	F6^i^—K2—F6^xvi^	105.87 (4)
F4^i^—Sb—F1^i^	42.52 (5)	F5^v^—K2—F6^xvi^	48.52 (4)
F5^ii^—Sb—F1^i^	93.92 (4)	F2^xiv^—K2—Sn^xvi^	80.82 (3)
F6—Sb—F1^i^	58.86 (4)	F2^xi^—K2—Sn^xvi^	75.59 (3)
F7^iii^—Sb—F1^i^	107.93 (3)	F3^v^—K2—Sn^xvi^	71.04 (4)
K1—Sb—F1^i^	147.81 (2)	F1—K2—Sn^xvi^	118.69 (4)
F6^iv^—Sb—F1^i^	107.05 (3)	F7^xv^—K2—Sn^xvi^	115.30 (4)
F5—Sb—F1^i^	82.00 (3)	F7^xvi^—K2—Sn^xvi^	29.81 (4)
F1—Sb—K1^v^	83.19 (5)	F6^i^—K2—Sn^xvi^	135.57 (4)
F3—Sb—K1^v^	42.71 (5)	F5^v^—K2—Sn^xvi^	30.19 (3)
F4—Sb—K1^v^	37.30 (4)	F6^xvi^—K2—Sn^xvi^	30.21 (2)
F2—Sb—K1^v^	120.02 (4)	F2^xiv^—K2—F4	134.72 (4)
F4^i^—Sb—K1^v^	104.20 (4)	F2^xi^—K2—F4	116.59 (4)
F5^ii^—Sb—K1^v^	160.47 (3)	F3^v^—K2—F4	70.89 (4)
F6—Sb—K1^v^	79.99 (3)	F1—K2—F4	43.32 (5)
F7^iii^—Sb—K1^v^	83.45 (3)	F7^xv^—K2—F4	63.76 (5)
K1—Sb—K1^v^	80.495 (13)	F7^xvi^—K2—F4	131.42 (5)
F6^iv^—Sb—K1^v^	152.92 (3)	F6^i^—K2—F4	80.07 (5)
F5—Sb—K1^v^	42.97 (2)	F5^v^—K2—F4	119.65 (4)
F1^i^—Sb—K1^v^	79.15 (2)	F6^xvi^—K2—F4	167.89 (4)
F1—Sb—K2^vi^	117.92 (5)	Sn^xvi^—K2—F4	141.35 (3)
F3—Sb—K2^vi^	75.50 (5)	F2^xiv^—K2—K1^xi^	103.45 (4)
F4—Sb—K2^vi^	146.86 (5)	F2^xi^—K2—K1^xi^	43.32 (3)
F2—Sb—K2^vi^	37.79 (4)	F3^v^—K2—K1^xi^	115.64 (4)
F4^i^—Sb—K2^vi^	140.23 (4)	F1—K2—K1^xi^	43.76 (3)
F5^ii^—Sb—K2^vi^	82.15 (3)	F7^xv^—K2—K1^xi^	128.37 (4)
F6—Sb—K2^vi^	84.58 (3)	F7^xvi^—K2—K1^xi^	145.82 (4)
F7^iii^—Sb—K2^vi^	42.62 (3)	F6^i^—K2—K1^xi^	45.47 (4)
K1—Sb—K2^vi^	72.944 (11)	F5^v^—K2—K1^xi^	93.55 (3)
F6^iv^—Sb—K2^vi^	43.58 (2)	F6^xvi^—K2—K1^xi^	101.07 (3)
F5—Sb—K2^vi^	84.66 (3)	Sn^xvi^—K2—K1^xi^	116.309 (16)
F1^i^—Sb—K2^vi^	138.75 (2)	F4—K2—K1^xi^	75.33 (3)
K1^v^—Sb—K2^vi^	115.096 (10)	F2^xiv^—K2—F3^xiv^	39.66 (4)
F1—Sb—Sb^i^	73.20 (5)	F2^xi^—K2—F3^xiv^	121.93 (4)
F3—Sb—Sb^i^	117.83 (5)	F3^v^—K2—F3^xiv^	106.25 (4)
F4—Sb—Sb^i^	39.94 (4)	F1—K2—F3^xiv^	155.67 (5)
F2—Sb—Sb^i^	147.66 (4)	F7^xv^—K2—F3^xiv^	50.88 (5)
F4^i^—Sb—Sb^i^	28.74 (4)	F7^xvi^—K2—F3^xiv^	59.33 (4)
F5^ii^—Sb—Sb^i^	89.16 (3)	F6^i^—K2—F3^xiv^	93.31 (4)
F6—Sb—Sb^i^	86.50 (3)	F5^v^—K2—F3^xiv^	113.77 (4)
F7^iii^—Sb—Sb^i^	134.11 (3)	F6^xvi^—K2—F3^xiv^	77.30 (4)
K1—Sb—Sb^i^	121.530 (9)	Sn^xvi^—K2—F3^xiv^	83.69 (2)
F6^iv^—Sb—Sb^i^	121.92 (2)	F4—K2—F3^xiv^	113.32 (4)
F5—Sb—Sb^i^	98.15 (3)	K1^xi^—K2—F3^xiv^	137.39 (2)
F1^i^—Sb—Sb^i^	28.39 (2)	F2^xiv^—K2—Sb^xiv^	28.40 (3)
K1^v^—Sb—Sb^i^	76.021 (8)	F2^xi^—K2—Sb^xiv^	106.25 (3)
K2^vi^—Sb—Sb^i^	164.150 (8)	F3^v^—K2—Sb^xiv^	132.62 (4)
F5—Sn—F5^iii^	180.00 (5)	F1—K2—Sb^xiv^	137.36 (3)
F5—Sn—F7^iii^	90.26 (7)	F7^xv^—K2—Sb^xiv^	59.99 (4)
F5^iii^—Sn—F7^iii^	89.74 (7)	F7^xvi^—K2—Sb^xiv^	84.57 (4)
F5—Sn—F7	89.74 (7)	F6^i^—K2—Sb^xiv^	64.94 (4)
F5^iii^—Sn—F7	90.26 (7)	F5^v^—K2—Sb^xiv^	131.15 (3)
F7^iii^—Sn—F7	180.00 (14)	F6^xvi^—K2—Sb^xiv^	84.19 (3)
F5—Sn—F6^iii^	91.07 (7)	Sn^xvi^—K2—Sb^xiv^	102.504 (11)
F5^iii^—Sn—F6^iii^	88.93 (7)	F4—K2—Sb^xiv^	107.92 (3)
F7^iii^—Sn—F6^iii^	88.84 (8)	K1^xi^—K2—Sb^xiv^	109.172 (12)
F7—Sn—F6^iii^	91.16 (8)	F3^xiv^—K2—Sb^xiv^	28.449 (19)
F5—Sn—F6	88.93 (7)	F2^xiv^—K2—K2^xvii^	41.75 (4)
F5^iii^—Sn—F6	91.07 (7)	F2^xi^—K2—K2^xvii^	41.53 (3)
F7^iii^—Sn—F6	91.16 (8)	F3^v^—K2—K2^xvii^	143.27 (4)
F7—Sn—F6	88.84 (8)	F1—K2—K2^xvii^	112.96 (4)
F6^iii^—Sn—F6	180.00 (16)	F7^xv^—K2—K2^xvii^	126.42 (4)
F5—Sn—K1^vii^	121.41 (5)	F7^xvi^—K2—K2^xvii^	88.06 (4)
F5^iii^—Sn—K1^vii^	58.59 (5)	F6^i^—K2—K2^xvii^	61.68 (4)
F7^iii^—Sn—K1^vii^	56.67 (6)	F5^v^—K2—K2^xvii^	82.59 (4)
F7—Sn—K1^vii^	123.33 (6)	F6^xvi^—K2—K2^xvii^	44.19 (3)
F6^iii^—Sn—K1^vii^	47.50 (6)	Sn^xvi^—K2—K2^xvii^	74.136 (15)
F6—Sn—K1^vii^	132.50 (6)	F4—K2—K2^xvii^	140.33 (3)
F5—Sn—K1^viii^	58.59 (5)	K1^xi^—K2—K2^xvii^	70.576 (15)
F5^iii^—Sn—K1^viii^	121.41 (5)	F3^xiv^—K2—K2^xvii^	80.83 (3)
F7^iii^—Sn—K1^viii^	123.33 (6)	Sb^xiv^—K2—K2^xvii^	66.445 (12)
F7—Sn—K1^viii^	56.67 (6)	F2^xiv^—K2—K1	149.45 (4)
F6^iii^—Sn—K1^viii^	132.50 (6)	F2^xi^—K2—K1	74.53 (3)
F6—Sn—K1^viii^	47.50 (6)	F3^v^—K2—K1	44.99 (4)
K1^vii^—Sn—K1^viii^	180.000 (14)	F1—K2—K1	46.84 (3)
F5—Sn—K2^ix^	129.80 (5)	F7^xv^—K2—K1	119.96 (4)
F5^iii^—Sn—K2^ix^	50.20 (5)	F7^xvi^—K2—K1	91.21 (4)
F7^iii^—Sn—K2^ix^	133.12 (5)	F6^i^—K2—K1	118.92 (4)
F7—Sn—K2^ix^	46.88 (5)	F5^v^—K2—K1	45.32 (3)
F6^iii^—Sn—K2^ix^	110.30 (6)	F6^xvi^—K2—K1	92.86 (3)
F6—Sn—K2^ix^	69.70 (6)	Sn^xvi^—K2—K1	73.573 (11)
K1^vii^—Sn—K2^ix^	105.549 (10)	F4—K2—K1	75.05 (3)
K1^viii^—Sn—K2^ix^	74.451 (10)	K1^xi^—K2—K1	74.327 (13)
F5—Sn—K2^v^	50.20 (5)	F3^xiv^—K2—K1	147.77 (2)
F5^iii^—Sn—K2^v^	129.80 (5)	Sb^xiv^—K2—K1	175.774 (15)
F7^iii^—Sn—K2^v^	46.88 (5)	K2^xvii^—K2—K1	113.33 (2)
F7—Sn—K2^v^	133.12 (5)	F2^xiv^—K2—K2^xviii^	75.81 (4)
F6^iii^—Sn—K2^v^	69.70 (6)	F2^xi^—K2—K2^xviii^	143.16 (4)
F6—Sn—K2^v^	110.30 (6)	F3^v^—K2—K2^xviii^	67.21 (4)
K1^vii^—Sn—K2^v^	74.451 (10)	F1—K2—K2^xviii^	135.04 (4)
K1^viii^—Sn—K2^v^	105.549 (10)	F7^xv^—K2—K2^xviii^	43.98 (3)
K2^ix^—Sn—K2^v^	180.000 (9)	F7^xvi^—K2—K2^xviii^	41.77 (4)
F5—Sn—Sb	65.81 (5)	F6^i^—K2—K2^xviii^	128.40 (5)
F5^iii^—Sn—Sb	114.19 (5)	F5^v^—K2—K2^xviii^	94.15 (4)
F7^iii^—Sn—Sb	56.42 (6)	F6^xvi^—K2—K2^xviii^	85.16 (3)
F7—Sn—Sb	123.58 (6)	Sn^xvi^—K2—K2^xviii^	71.414 (13)
F6^iii^—Sn—Sb	136.06 (6)	F4—K2—K2^xviii^	99.52 (3)
F6—Sn—Sb	43.94 (6)	K1^xi^—K2—K2^xviii^	172.16 (2)
K1^vii^—Sn—Sb	112.591 (9)	F3^xiv^—K2—K2^xviii^	39.05 (2)
K1^viii^—Sn—Sb	67.409 (9)	Sb^xiv^—K2—K2^xviii^	66.425 (12)
K2^ix^—Sn—Sb	113.168 (8)	K2^xvii^—K2—K2^xviii^	111.90 (2)
K2^v^—Sn—Sb	66.832 (8)	K1—K2—K2^xviii^	110.379 (17)
F5—Sn—Sb^iii^	114.19 (5)	F2^xiv^—K2—F1^xi^	120.78 (4)
F5^iii^—Sn—Sb^iii^	65.81 (5)	F2^xi^—K2—F1^xi^	37.66 (4)
F7^iii^—Sn—Sb^iii^	123.58 (6)	F3^v^—K2—F1^xi^	83.92 (4)
F7—Sn—Sb^iii^	56.42 (6)	F1—K2—F1^xi^	46.14 (5)
F6^iii^—Sn—Sb^iii^	43.94 (6)	F7^xv^—K2—F1^xi^	151.93 (5)
F6—Sn—Sb^iii^	136.06 (6)	F7^xvi^—K2—F1^xi^	108.92 (4)
K1^vii^—Sn—Sb^iii^	67.409 (9)	F6^i^—K2—F1^xi^	86.80 (5)
K1^viii^—Sn—Sb^iii^	112.591 (9)	F5^v^—K2—F1^xi^	52.66 (4)
K2^ix^—Sn—Sb^iii^	66.832 (8)	F6^xvi^—K2—F1^xi^	80.65 (4)
K2^v^—Sn—Sb^iii^	113.168 (8)	Sn^xvi^—K2—F1^xi^	80.33 (2)
Sb—Sn—Sb^iii^	180.0	F4—K2—F1^xi^	89.28 (3)
F4^v^—K1—F2	135.92 (6)	K1^xi^—K2—F1^xi^	42.39 (2)
F4^v^—K1—F1^xi^	148.39 (5)	F3^xiv^—K2—F1^xi^	157.10 (4)
F2—K1—F1^xi^	75.39 (5)	Sb^xiv^—K2—F1^xi^	142.70 (2)
F4^v^—K1—F6^xii^	112.69 (5)	K2^xvii^—K2—F1^xi^	79.10 (3)
F2—K1—F6^xii^	69.51 (5)	K1—K2—F1^xi^	39.05 (2)
F1^xi^—K1—F6^xii^	78.81 (5)	K2^xviii^—K2—F1^xi^	144.60 (3)
F4^v^—K1—F3^v^	56.26 (5)	F2^xiv^—K2—K1^v^	132.57 (4)
F2—K1—F3^v^	111.27 (5)	F2^xi^—K2—K1^v^	143.70 (4)
F1^xi^—K1—F3^v^	115.30 (5)	F3^v^—K2—K1^v^	43.33 (4)
F6^xii^—K1—F3^v^	165.82 (5)	F1—K2—K1^v^	69.71 (4)
F4^v^—K1—F5^v^	77.12 (5)	F7^xv^—K2—K1^v^	47.07 (4)
F2—K1—F5^v^	138.76 (5)	F7^xvi^—K2—K1^v^	94.17 (4)
F1^xi^—K1—F5^v^	73.05 (5)	F6^i^—K2—K1^v^	113.54 (4)
F6^xii^—K1—F5^v^	127.69 (6)	F5^v^—K2—K1^v^	104.38 (4)
F3^v^—K1—F5^v^	61.68 (5)	F6^xvi^—K2—K1^v^	140.15 (3)
F4^v^—K1—F1	125.45 (5)	Sn^xvi^—K2—K1^v^	110.896 (12)
F2—K1—F1	54.76 (4)	F4—K2—K1^v^	37.32 (2)
F1^xi^—K1—F1	63.69 (6)	K1^xi^—K2—K1^v^	110.414 (15)
F6^xii^—K1—F1	117.92 (5)	F3^xiv^—K2—K1^v^	94.36 (3)
F3^v^—K1—F1	70.23 (4)	Sb^xiv^—K2—K1^v^	106.861 (15)
F5^v^—K1—F1	87.34 (4)	K2^xvii^—K2—K1^v^	172.75 (2)
F4^v^—K1—F3	89.08 (5)	K1—K2—K1^v^	73.558 (15)
F2—K1—F3	53.71 (5)	K2^xviii^—K2—K1^v^	66.149 (15)
F1^xi^—K1—F3	114.74 (4)	F1^xi^—K2—K1^v^	106.60 (2)
F6^xii^—K1—F3	111.78 (5)	F2^xiv^—K2—Sb^xi^	96.86 (4)
F3^v^—K1—F3	62.17 (5)	F2^xi^—K2—Sb^xi^	20.08 (3)
F5^v^—K1—F3	119.86 (5)	F3^v^—K2—Sb^xi^	96.64 (4)
F1—K1—F3	54.61 (4)	F1—K2—Sb^xi^	71.72 (4)
F4^v^—K1—F4^xii^	97.18 (5)	F7^xv^—K2—Sb^xi^	176.66 (4)
F2—K1—F4^xii^	121.36 (5)	F7^xvi^—K2—Sb^xi^	93.17 (4)
F1^xi^—K1—F4^xii^	58.73 (4)	F6^i^—K2—Sb^xi^	87.26 (4)
F6^xii^—K1—F4^xii^	67.58 (5)	F5^v^—K2—Sb^xi^	44.16 (3)
F3^v^—K1—F4^xii^	120.01 (5)	F6^xvi^—K2—Sb^xi^	55.73 (3)
F5^v^—K1—F4^xii^	60.16 (5)	Sn^xvi^—K2—Sb^xi^	63.440 (9)
F1—K1—F4^xii^	119.49 (5)	F4—K2—Sb^xi^	115.04 (3)
F3—K1—F4^xii^	173.45 (4)	K1^xi^—K2—Sb^xi^	52.902 (10)
F4^v^—K1—F7^xii^	77.31 (5)	F3^xiv^—K2—Sb^xi^	130.95 (3)
F2—K1—F7^xii^	69.12 (4)	Sb^xiv^—K2—Sb^xi^	123.080 (12)
F1^xi^—K1—F7^xii^	128.67 (5)	K2^xvii^—K2—Sb^xi^	56.635 (14)
F6^xii^—K1—F7^xii^	54.82 (5)	K1—K2—Sb^xi^	56.866 (11)
F3^v^—K1—F7^xii^	111.57 (5)	K2^xviii^—K2—Sb^xi^	134.85 (2)
F5^v^—K1—F7^xii^	152.00 (5)	F1^xi^—K2—Sb^xi^	26.15 (2)
F1—K1—F7^xii^	117.02 (4)	K1^v^—K2—Sb^xi^	129.978 (13)
F3—K1—F7^xii^	70.83 (4)	Sb—F1—K2	140.58 (8)
F4^xii^—K1—F7^xii^	112.37 (5)	Sb—F1—K1^xi^	117.20 (6)
F4^v^—K1—F5^xii^	59.59 (5)	K2—F1—K1^xi^	92.59 (5)
F2—K1—F5^xii^	113.82 (4)	Sb—F1—K1	97.72 (5)
F1^xi^—K1—F5^xii^	118.46 (5)	K2—F1—K1	90.44 (4)
F6^xii^—K1—F5^xii^	54.02 (4)	K1^xi^—F1—K1	116.31 (6)
F3^v^—K1—F5^xii^	115.85 (4)	Sb—F1—Sb^i^	78.40 (5)
F5^v^—K1—F5^xii^	104.33 (4)	K2—F1—Sb^i^	81.29 (4)
F1—K1—F5^xii^	168.30 (4)	K1^xi^—F1—Sb^i^	81.82 (3)
F3—K1—F5^xii^	118.02 (5)	K1—F1—Sb^i^	160.56 (6)
F4^xii^—K1—F5^xii^	67.26 (5)	Sb—F1—K2^xi^	83.58 (5)
F7^xii^—K1—F5^xii^	51.96 (4)	K2—F1—K2^xi^	133.86 (5)
F4^v^—K1—K1^xiii^	52.74 (4)	K1^xi^—F1—K2^xi^	67.96 (4)
F2—K1—K1^xiii^	157.57 (4)	K1—F1—K2^xi^	65.15 (3)
F1^xi^—K1—K1^xiii^	100.64 (4)	Sb^i^—F1—K2^xi^	132.27 (4)
F6^xii^—K1—K1^xiii^	88.06 (4)	Sb—F1—Sb^xi^	145.08 (7)
F3^v^—K1—K1^xiii^	90.53 (3)	K2—F1—Sb^xi^	71.96 (3)
F5^v^—K1—K1^xiii^	56.19 (4)	K1^xi^—F1—Sb^xi^	57.94 (3)
F1—K1—K1^xiii^	143.52 (4)	K1—F1—Sb^xi^	63.04 (3)
F3—K1—K1^xiii^	141.78 (4)	Sb^i^—F1—Sb^xi^	129.25 (3)
F4^xii^—K1—K1^xiii^	44.44 (3)	K2^xi^—F1—Sb^xi^	62.18 (3)
F7^xii^—K1—K1^xiii^	98.64 (3)	Sb—F2—K2^vi^	113.81 (7)
F5^xii^—K1—K1^xiii^	48.14 (3)	Sb—F2—K2^xi^	133.55 (7)
F4^v^—K1—Sb	119.75 (5)	K2^vi^—F2—K2^xi^	96.72 (5)
F2—K1—Sb	33.04 (3)	Sb—F2—K1	101.44 (6)
F1^xi^—K1—Sb	83.66 (3)	K2^vi^—F2—K1	117.00 (5)
F6^xii^—K1—Sb	102.46 (4)	K2^xi^—F2—K1	93.98 (5)
F3^v^—K1—Sb	78.76 (3)	Sb—F3—K2^v^	144.17 (8)
F5^v^—K1—Sb	116.80 (4)	Sb—F3—K1^v^	109.46 (6)
F1—K1—Sb	31.07 (3)	K2^v^—F3—K1^v^	92.49 (5)
F3—K1—Sb	31.44 (3)	Sb—F3—K1	96.64 (6)
F4^xii^—K1—Sb	142.03 (3)	K2^v^—F3—K1	97.51 (5)
F7^xii^—K1—Sb	85.99 (3)	K1^v^—F3—K1	117.83 (5)
F5^xii^—K1—Sb	137.87 (3)	Sb—F3—K2^vi^	76.06 (5)
K1^xiii^—K1—Sb	169.27 (2)	K2^v^—F3—K2^vi^	73.75 (4)
F4^v^—K1—Sn^xii^	83.78 (4)	K1^v^—F3—K2^vi^	158.09 (6)
F2—K1—Sn^xii^	82.30 (3)	K1—F3—K2^vi^	81.50 (4)
F1^xi^—K1—Sn^xii^	109.42 (4)	Sb—F3—Sb^v^	140.22 (6)
F6^xii^—K1—Sn^xii^	30.96 (3)	K2^v^—F3—Sb^v^	75.23 (3)
F3^v^—K1—Sn^xii^	135.18 (3)	K1^v^—F3—Sb^v^	59.92 (3)
F5^v^—K1—Sn^xii^	133.25 (4)	K1—F3—Sb^v^	63.99 (3)
F1—K1—Sn^xii^	137.06 (3)	K2^vi^—F3—Sb^v^	129.27 (4)
F3—K1—Sn^xii^	101.81 (3)	Sb—F4—K1^v^	114.26 (6)
F4^xii^—K1—Sn^xii^	80.88 (3)	Sb—F4—Sb^i^	111.31 (6)
F7^xii^—K1—Sn^xii^	31.65 (3)	K1^v^—F4—Sb^i^	130.88 (7)
F5^xii^—K1—Sn^xii^	31.61 (3)	Sb—F4—K1^viii^	121.86 (8)
K1^xiii^—K1—Sn^xii^	78.252 (16)	K1^v^—F4—K1^viii^	82.82 (5)
Sb—K1—Sn^xii^	109.774 (12)	Sb^i^—F4—K1^viii^	88.45 (5)
F4^v^—K1—K2^xi^	159.66 (4)	Sb—F4—K2	90.97 (6)
F2—K1—K2^xi^	42.70 (4)	K1^v^—F4—K2	79.93 (4)
F1^xi^—K1—K2^xi^	43.65 (3)	Sb^i^—F4—K2	81.98 (4)
F6^xii^—K1—K2^xi^	47.22 (3)	K1^viii^—F4—K2	146.97 (6)
F3^v^—K1—K2^xi^	142.68 (3)	Sn—F5—K1^v^	154.71 (8)
F5^v^—K1—K2^xi^	116.36 (4)	Sn—F5—K2^v^	99.61 (6)
F1—K1—K2^xi^	72.46 (3)	K1^v^—F5—K2^v^	86.58 (5)
F3—K1—K2^xi^	96.07 (3)	Sn—F5—Sb^x^	118.58 (7)
F4^xii^—K1—K2^xi^	78.71 (3)	K1^v^—F5—Sb^x^	85.29 (4)
F7^xii^—K1—K2^xi^	85.80 (4)	K2^v^—F5—Sb^x^	92.70 (4)
F5^xii^—K1—K2^xi^	100.98 (3)	Sn—F5—K1^viii^	89.79 (5)
K1^xiii^—K1—K2^xi^	120.30 (2)	K1^v^—F5—K1^viii^	75.67 (4)
Sb—K1—K2^xi^	69.495 (11)	K2^v^—F5—K1^viii^	154.85 (7)
Sn^xii^—K1—K2^xi^	75.912 (12)	Sb^x^—F5—K1^viii^	103.25 (5)
F4^v^—K1—Sb^v^	28.43 (4)	Sn—F5—Sb	86.58 (6)
F2—K1—Sb^v^	127.52 (4)	K1^v^—F5—Sb	70.26 (4)
F1^xi^—K1—Sb^v^	136.40 (3)	K2^v^—F5—Sb	79.97 (4)
F6^xii^—K1—Sb^v^	140.46 (3)	Sb^x^—F5—Sb	154.74 (5)
F3^v^—K1—Sb^v^	27.83 (3)	K1^viii^—F5—Sb	77.35 (4)
F5^v^—K1—Sb^v^	66.77 (4)	Sn—F5—Sb^xv^	124.19 (7)
F1—K1—Sb^v^	97.55 (3)	K1^v^—F5—Sb^xv^	61.95 (3)
F3—K1—Sb^v^	73.82 (3)	K2^v^—F5—Sb^xv^	132.77 (4)
F4^xii^—K1—Sb^v^	111.02 (3)	Sb^x^—F5—Sb^xv^	53.18 (3)
F7^xii^—K1—Sb^v^	94.88 (4)	K1^viii^—F5—Sb^xv^	52.27 (3)
F5^xii^—K1—Sb^v^	88.02 (3)	Sb—F5—Sb^xv^	116.05 (4)
K1^xiii^—K1—Sb^v^	70.564 (14)	Sn—F6—K1^viii^	101.55 (7)
Sb—K1—Sb^v^	99.505 (13)	Sn—F6—K2^i^	152.63 (9)
Sn^xii^—K1—Sb^v^	110.137 (11)	K1^viii^—F6—K2^i^	87.31 (5)
K2^xi^—K1—Sb^v^	168.929 (17)	Sn—F6—Sb	110.18 (7)
F4^v^—K1—Sb^xi^	122.89 (4)	K1^viii^—F6—Sb	96.60 (5)
F2—K1—Sb^xi^	100.69 (4)	K2^i^—F6—Sb	94.19 (5)
F1^xi^—K1—Sb^xi^	25.51 (3)	Sn—F6—K2^ix^	80.09 (6)
F6^xii^—K1—Sb^xi^	90.54 (4)	K1^viii^—F6—K2^ix^	89.28 (6)
F3^v^—K1—Sb^xi^	103.00 (3)	K2^i^—F6—K2^ix^	74.13 (4)
F5^v^—K1—Sb^xi^	49.35 (4)	Sb—F6—K2^ix^	166.72 (6)
F1—K1—Sb^xi^	76.30 (3)	Sn—F6—Sb^iv^	91.99 (6)
F3—K1—Sb^xi^	130.90 (3)	K1^viii^—F6—Sb^iv^	154.48 (6)
F4^xii^—K1—Sb^xi^	43.41 (3)	K2^i^—F6—Sb^iv^	71.48 (4)
F7^xii^—K1—Sb^xi^	145.36 (4)	Sb—F6—Sb^iv^	98.89 (5)
F5^xii^—K1—Sb^xi^	110.56 (4)	K2^ix^—F6—Sb^iv^	71.69 (3)
K1^xiii^—K1—Sb^xi^	78.484 (17)	Sn—F6—Sbi	142.88 (7)
Sb—K1—Sb^xi^	103.188 (10)	K1i—F6—Sbi	54.56 (3)
Sn^xii^—K1—Sb^xi^	116.563 (15)	K2i—F6—Sbi	62.34 (3)
K2^xi^—K1—Sb^xi^	67.191 (11)	Sb—F6—Sbi	54.21 (3)
Sb^v^—K1—Sb^xi^	115.843 (12)	K2i—F6—Sbi	122.30 (4)
F4^v^—K1—K2	94.67 (4)	Sbi—F6—Sbi	121.86 (4)
F2—K1—K2	97.42 (3)	Sn—F6—Sbi	110.68 (7)
F1^xi^—K1—K2	72.99 (4)	K1i—F6—Sbi	45.15 (3)
F6^xii^—K1—K2	151.21 (4)	K2i—F6—Sbi	58.40 (4)
F3^v^—K1—K2	42.51 (3)	Sb—F6—Sbi	128.37 (5)
F5^v^—K1—K2	48.10 (4)	K2i—F6—Sbi	50.61 (3)
F1—K1—K2	42.72 (3)	Sbi—F6—Sbi	109.80 (4)
F3—K1—K2	76.00 (3)	Sbi—F6—Sbi	74.17 (2)
F4^xii^—K1—K2	101.45 (4)	Sn—F7—K2i	160.99 (8)
F7^xii^—K1—K2	145.89 (4)	Sn—F7—K2i	103.30 (7)
F5^xii^—K1—K2	148.35 (3)	K2i—F7—K2i	94.25 (5)
K1^xiii^—K1—K2	102.511 (19)	Sn—F7—K1i	91.68 (7)
Sb—K1—K2	69.152 (11)	K2i—F7—K1i	92.57 (5)
Sn^xii^—K1—K2	177.363 (18)	K2i—F7—K1i	99.48 (5)
K2^xi^—K1—K2	105.673 (13)	Sn—F7—Sbi	96.03 (6)
Sb^v^—K1—K2	67.947 (11)	K2i—F7—Sbi	77.39 (5)
Sb^xi^—K1—K2	66.072 (11)	K2i—F7—Sbi	87.21 (5)
F4^v^—K1—K2^v^	62.75 (4)	K1i—F7—Sbi	168.39 (6)
F2—K1—K2^v^	73.17 (4)	Sn—F7—Sbi	84.28 (5)
F1^xi^—K1—K2^v^	148.20 (3)	K2i—F7—Sbi	77.70 (4)
F6^xii^—K1—K2^v^	94.45 (4)	K2i—F7—Sbi	171.22 (6)
F3^v^—K1—K2^v^	72.82 (3)	K1i—F7—Sbi	84.54 (4)
F5^v^—K1—K2^v^	131.10 (4)	Sbi—F7—Sbi	87.60 (4)
F1—K1—K2^v^	93.76 (3)	Sn—F7—Sbi	128.27 (7)
F3—K1—K2^v^	39.16 (3)	K2i—F7—Sbi	67.72 (3)
F4^xii^—K1—K2^v^	146.51 (3)	K2i—F7—Sbi	57.73 (3)
F7^xii^—K1—K2^v^	40.37 (4)	K1i—F7—Sbi	52.36 (3)
F5^xii^—K1—K2^v^	79.29 (4)	Sbi—F7—Sbi	126.52 (4)
K1^xiii^—K1—K2^v^	110.234 (19)	Sbi—F7—Sbi	120.88 (4)
Sb—K1—K2^v^	67.341 (11)		

Symmetry codes: (i) −*x*+1, −*y*, −*z*+1; (ii) *x*−1, *y*, *z*; (iii) −*x*+2, −*y*, −*z*; (iv) −*x*+1, −*y*, −*z*; (v) −*x*+2, −*y*+1, −*z*+1; (vi) *x*, *y*, *z*−1; (vii) −*x*+2, −*y*+1, −*z*; (viii) *x*, *y*−1, *z*; (ix) *x*, *y*−1, *z*−1; (x) *x*+1, *y*, *z*; (xi) −*x*+1, −*y*+1, −*z*+1; (xii) *x*, *y*+1, *z*; (xiii) −*x*+2, −*y*+2, −*z*+1; (xiv) *x*, *y*, *z*+1; (xv) −*x*+2, −*y*, −*z*+1; (xvi) *x*, *y*+1, *z*+1; (xvii) −*x*+1, −*y*+1, −*z*+2; (xviii) −*x*+2, −*y*+1, −*z*+2.
